# Changes in Simple Spirometric Parameters After Lobectomy for Bronchial Carcinoma

**DOI:** 10.15171/jcvtr.2015.15

**Published:** 2015

**Authors:** Eleni Drakou, Meletios A. Kanakis, Lila Papadimitriou, Nicoletta Iacovidou, Nikolaos Vrachnis, Stefanos Nicolouzos, Constantinos Loukas, Achilleas Lioulias

**Affiliations:** ^1^ Department of Thoracic Surgery, Sismanoglio General Hospital of Athens, Athens, Greece; ^2^ School of Medicine, University of Athens, Athens, Greece; ^3^ Department of Neonatology, Aretaieio Hospital, School of Medicine, University of Athens, Athens, Greece; ^4^ 2nd Department of Obstetrics and Gynecology, Aretaieio Hospital, School of Medicine, University of Athens, Athens, Greece; ^5^ Medical Physics Laboratory, School of Medicine, University of Athens, Athens, Greece

**Keywords:** FEV1, FVC, Lung Cancer, Lung Function, Spirometry, Thoracotomy

## Abstract

***Introduction:*** The purpose of this study was to describe the postoperative changes in lung function after pure open lobectomy for lung carcinoma.

***Methods:*** 30 patients (mean age 64 ± 7 years old, 16 men and 14 women) underwent a left or right lobectomy. They underwent spirometric pulmonary tests preoperatively, and at 1 and 6 months after the operation.

***Results:*** The average preoperative forced expiratory volume in 1 second (FEV1) was 2.55±0.62lt and the mean postoperative FEV1 at 1 and 6 months was 1.97 ± 0.59 L and 2.15±0.66 L respectively. The percentage losses for FEV1 were 22.7% and 15.4% after 1 and 6 months respectively. An average percentage increase of 9.4% for FEV1 was estimated at the time of 6 months in comparison with this of 1 month after the operation. The average preoperative forced vital capacity (FVC) was 3.17 ± 0.81 L and the mean postoperative FVC at 1 and 6 months after the operation was 2.50 ± 0.63 L and 2.72 ± 0.67 L respectively. The percentage losses for FVC were 21.1% and 14.2% after 1 and 6 months respectively. An average percentage increase of 8.7% was observed at the time period of 6 months in comparison with this of 1 month after the operation.

***Conclusion:*** Although, we observed a significant decrease in FEV1 and FVC after the operation, all patients were in excellent clinical status. FEV1 and FVC of 6 months were increased in comparison with the respective values of 1 month after the operation, but did not reach the preoperative values in any patient.

## Introduction

Lung resection offers the best prospective for long-term survival in patients with nonmetastatic bronchogenic carcinoma.^1^ However, the removal of lung parenchyma from patients with lung carcinoma, who are usually smokers with an already compromised pulmonary status, may lead to respiratory failure. For these reasons, it is imperative to determine the operability of these patients in order to predict residual respiratory function after surgery. Operation could be performed with safety when preoperative forced expiratory volume in 1 second (FEV1) is greater than 2.0 L or 60% of predicted and there is a diffusion capacity above 60% of predicted.^2,3^ The British Thoracic Society guidelines, suggest that a mortality rate of <5% can be achieved if the preoperative FEV1 is >1.5lt and >2lt for a lobectomy and pneumonectomy respectively.^4^ Measurements of these alterations after pneumonectomy and lobectomy vary between studies in English literature. With the present study we aimed at to describing the postoperative changes in lung function after pure open lobectomy for lung carcinoma.

## Patients and Methods

All patients enrolled in this study were referred to the Thoracic Department for treatment of proven lung cancer. 30 patients (mean age 64 ± 7 years old, 16 men and 14 women) underwent a pure left or right lobectomy (right upper lobectomy in 8 patients, left upper lobectomy in 7 patients, right lower lobectomy in 7 patients, left lower lobectomy in 6 patients and middle lobectomy in 2 patients). All patients suffered from non-small cell lung cancer and had clearly an indication for operative treatment. Postoperatively, 4 patients were in clinical stage IB, 22 in stage IB, and 4 in stage IIB. Histologically there were 20 squamous cell carcinomas, 9 adenocarcinomas and 1 patient had bronchoalveolar carcinoma. None of the patients received preoperative induction therapy and all of them received postoperatively (one month after the operation) chemotherapy.

All patients were smokers before the operation and all of them stopped smoking after the operation and during the study period. They underwent spirometric pulmonary tests preoperatively, and at 1 and 6 months after the operation. Pulmonary function tests were performed by spirometry while the patient was at rest and in a seated in the upright position. Spirometry tests were performed by the same examiner using Cosmed pony spirometer (Cosmed Srl, Roma Italy). Of all recorded parameters the following two were used for the assessment of operability: the best FEV1 and the best forced vital capacity (FVC). All lobectomies were performed with an open posterolateral thoracotomy. The same team of thoracic surgeons performed all operations. Patients with chronic obstructive pulmonary disease (COPD, athma) or chronic interstitial disease, heart disease, chest wall deformities, locally advanced tumors and these who had undergone radiotherapy as patients receiving beta-blockers were excluded from the study.

Preoperatively, all patients guided by the same physiotherapist and at first postoperative day they participated in the standard physiotherapy protocol. The patients received bronchodilators only at the first 3 postoperative days.

Postoperative management included local blockade with ropivacaine 0.5% two levels above and below surgical incision at the operating room and intravenous systematic use of acetaminophen (1 g, q6h for 24 hours) and meperidine (0.5 mg/kg, q6h for 24 hours) alternately for the first two postoperative days. At third postoperative day there was systematic use of per os acetaminophen (1 g, q6h for 24 hours), which it was continued for the first 15 days after discharge from hospital. All patients did not report remarkable pain during the examination.

## Statistical Analysis

Statistical analysis between preoperative and postoperative variables was performed by Wilcoxon test. A *P*-value less than 0.05 was accepted as the significance limit. The choice of at least ten subjects per group was based on a two-tailed test, with α =.05 and power (1-b)= 0.80.^5^ Descriptive statistics (mean and standard deviation) for FEV1 and FVC were calculated before and after the intervention.

## Results

All patients tolerated well the procedure and none reported symptom of dyspnea during the study period. The mean spirometric values of patients preoperatively and at 1 and 6-months after lobectomy is shown in Table 1.

**Table 1:** Mean ±SD Values of FEV1 and FVC in Studied Patients

**Study Period**	**FEV1 (L)**	**FVC (L)**
preoperatively	2.55 ± 0.66	3.17 ± 0.81
1 month after operation	1.97 ± 0.59	2.50 ± 0.63
6 months after operation	2.15 ± 0.62	2.72 ± 0.67

Abbreviations: FEV1, forced expiratory volume in 1 ; FVC, forced vital capacity.

**Figure 1:**
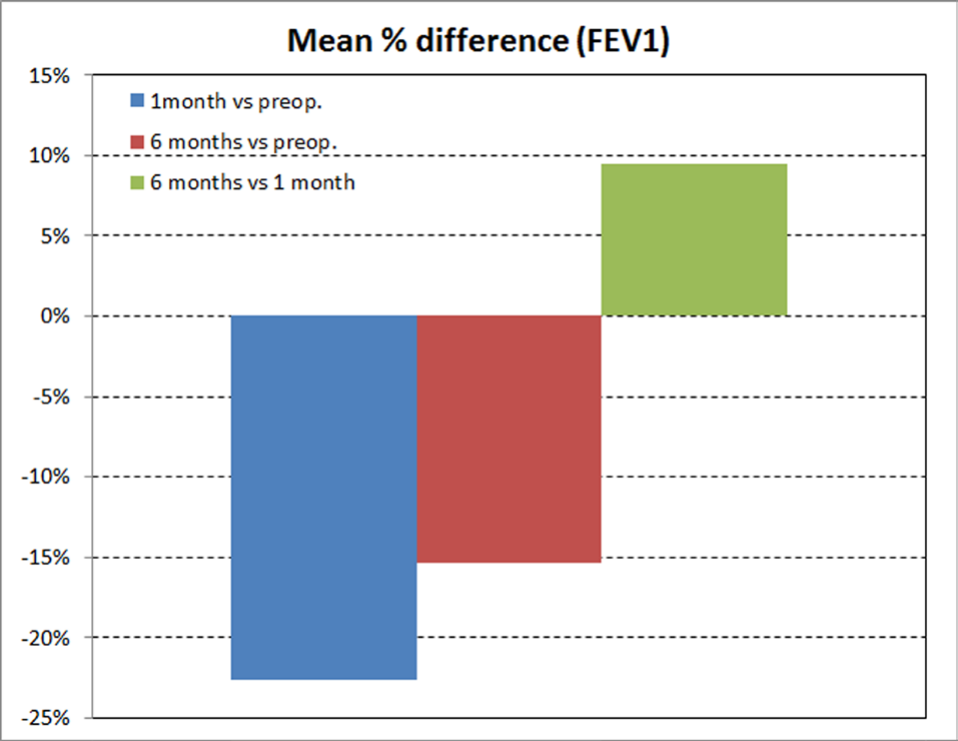
Schematic Representation Depicting the Average Percentage Variations for FEV1 in the Different Studied Periods.

The average preoperative FEV 1 was 2.55 ± 0.62 L and the mean postoperative FEV1 at 1 and 6 months was 1.97 ± 0.59 L and 2.15 ± 0.66 L respectively. Therefore FEV1 decreased significantly 1 month after operation and improved after 6 months, but remained in lower levels compared with the preoperative values (Table 2). The percentage losses for FEV1 were 22.7% and 15.4% after 1 and 6 months respectively. An average percentage increase of 9.4% for FEV1 was estimated at the time of 6 months in comparison with this of 1 month after the operation (Figure 1).

**Table 2:** Comparison of FEV1 and FVC in the Different Studied Periods

**Study period**	**FEV1**	**FVC**
Preoperatively vs 1 month	*P *< .05	*P *< .05
Preoperatively vs 6 months	*P *< .05	*P *< .05
1 month vs 6 months	Nonsignificant	Nonsignificant

Abbreviations: FEV1, forced expiratory volume in 1 ; FVC, forced vital capacity.

**Figure 2:**
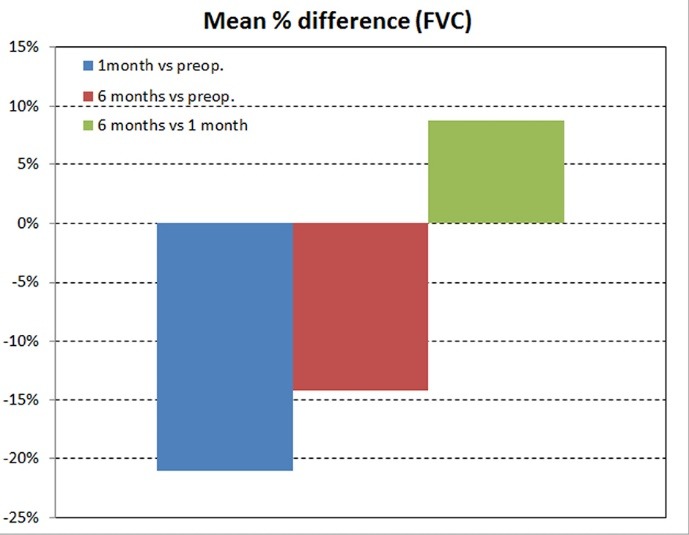
Schematic Representation Depicting the Average Percentage Variations for FVC in the Different Studied Periods.

The average preoperative FVC was 3.17 ± 0.81 L and the mean postoperative FVC at 1 and 6 months after the operation was 2.50 ± 0.63 L and 2.72 ± 0.67 L respectively. Therefore FVC decreased significantly 1 month after operation and improved after 6 months, but remained in decreased levels compared with the preoperative values (Table 2). The percentage losses for FVC were 21.1% and 14.2% after 1 and 6 months respectively. An average percentage increase of 8.7% was observed at the time period of 6 months in comparison with this of 1 month after the operation (Figure 2).

A schematic representation of these spirometric changes is depicted in Figures 1 and 2.

All patients were in an excellent clinical status and they observed a progressive improvement to their stamina, 6 months after the operation.

## Discussion

Measurements of postoperative spirometric values after lung resection including pneumonectomy, lobectomy, bilobectomy and segmentectomy vary. Regarding pneumonectomy, a deterioration in FEV1 of 29% to 35% and in FVC of 27% to 44% has been reported among studies.^6-10^ After lobectomy the results are more varied with a fall in FEV1 of 12% to 23% and in FVC of 10% to 30%.^7,9,11^ Additionally, in cases, that segmental resection could be performed; postoperative lung function seems to be preserved in comparison with lobectomy.^12^ These differenced can be explained by variations in methods, underlying disease and certainly time of analysis after resection.^6^ Baltayiannis et al observed a decrease in FEV1 and FVC, 3 months after the operation and slightly improvement at 6 months.^13^

Decrease in FEV1 and FVC are less than could be estimated from the number of resected segments after lobectomy, which is easily explained as the pre-existed neoplasm already causes a reduction in lung function. This is also confirmed by scintigraphy studies.^14^ Many equations have been proposed for prediction of postoperative FEV1 and FVC after lung resection. Juhl and Frost have proposed: Predicted postoperative FEV1 = preoperative FEV1 × [1-(S × 0.0526)] and predicted postoperative FVC = preoperative FVC × [1-(S × 0.0526)], where S is the number of resected segments.^15^ However, in some cases the results of the formula may be disappointing for its ability to predict postoperative pulmonary function.^16^ Zeicher et al^17^ demonstrated that simple calculation based on equation of Juhl and Frost systematically underestimated the actual postoperative FEV1 for patients undergoing lobectomy by 250 ml. Baltayiannis et al proposed a more precise equation for FEV1 after lobectomy, which can be derived from the following equation: FEV1 postoperative = 0.00211 + 0.896660 × FEV1 preoperative.^13^

It is generally accepted that with these aforementioned equations and others a postoperative estimation of FEV1 and FVC can be achieved, however the purpose of this study was not to test the reliability of these calculations in our patients. We tried to compare the postoperative changes in spirometric values after simple lobectomy at certain time points after the operation. Although Larsen et al did not observe significant differences between patients having simple lobectomy or bilobectomy, we tried to analyze simple lobectomy by the classic open approach (thoracotomy) in order to exclude any bias. Moreover, lobectomy may improve pulmonary function in patients suffering from COPD, so this group of patients was totally excluded from this study.^18,19^ All studied patients had a diagnosis of bronchogenic carcinoma and tumors that would invade chest wall, diaphragm, phrenic nerve or trachea were also excluded from the study as these tumors may affect the respiratory status indirectly. In addition for similar reasons, patients who had received radiotherapy or those having cardiac problems or with chest wall deformities were also excluded. Postoperative pain and muscle spasm are well described causes of impairment of the chest wall function and subsequently decrease of lung volume.^20,21^ It is a common clinical experience that this is prominent at the first postoperative days. The spirometry was performed at 1 and 6 months after the operation. In this study, all patients declared that they did not feel pain that would prevent them performing the examination. In addition, all of them had stopped smoking after surgery.

## Conclusion

Despite the limited number of patients, this study attempted at including patients that had simple lobectomy without comorbidities or other factors that could additionally influence their respiratory status. In this group of patients, lobectomy is associated with minor deterioration of lung function as this was calculated by spirometry. Although, we observed a significant decrease in FEV1 and FVC after the operation, all patients were in excellent clinical status and FEV1 and FVC of 6 months were increased in comparison with the respective values of 1 month after the operation, but did not reach the preoperative values in any patient.

## Ethical Issues

The study was approved by the Hospital Bioethical Committee and informed consent was obtained from all patients.

## Conflict of Interests

The authors declare no conflict of interests.
